# Celastrol suppresses human pancreatic cancer via m^6^A-YTHDF3-mediated downregulation of Claspin and Bcl-2

**DOI:** 10.1007/s12672-023-00838-5

**Published:** 2023-12-18

**Authors:** Yang Zhou, Haoran Zhuang, Yuxiang Liu, Jing Yin, Xiaoying Wei, Yue Qiu, Zhen Tian, Tingyu Miao, Jing Chen, Peifen Li, Xiao Xu, Wenjuan Wu, Huanan Li, Weigan Shen

**Affiliations:** 1 Department of Cell Biology, School of Medicine of Yangzhou University, Yangzhou, Jiangsu China; 2 Department of Oncology, Taizhou Hospital of Traditional Chinese Medicine, Taizhou, Jiangsu China; 3 Department of Oncology, Clinical Medical College, Yangzhou University, Yangzhou, Jiangsu China; 4 Jiangsu Key Laboratory of Integrated Traditional Chinese and Western Medicine for Prevention and Treatment of Senile Diseases, Yangzhou University, Yangzhou, Jiangsu China

**Keywords:** Celastrol, RNA m^6^A modification, Pancreatic cancer, Claspin

## Abstract

**Background:**

Celastrol has been revealed to exhibit anticancer pharmacological activity, however, the molecular mechanisms of celastrol involved in pancreatic cancer remain to be further elucidated. The present study was to illustrate whether celastrol suppresses pancreatic cancer through modulating RNA m^6^A modification.

**Methods:**

Effect of celastrol treatment on the malignant phenotypes of pancreatic cancer cells was evaluated by CCK-8 assay, EdU assay, colony formation assay, flow cytometry analysis and subcutaneous xenograft experiments. RNA sequencing (RNA-seq) analysis was carried out to analyze the genes differentially expressed in celastrol-treated pancreatic cancer cells. RT-qPCR, Western blotting and immunohistochemistry were employed to evaluate the expression of the indicated genes. RNA dot blot and quantification of total RNA m^6^A modification assays, MeRIP-qPCR assay, RIP-qPCR assay, RNA stability and protein stability assays were applied to evaluate the regulatory mechanism of celastrol treatment in pancreatic cancer cells.

**Results:**

We demonstrated that celastrol suppressed cell proliferation and induced cell cycle arrest and apoptosis of pancreatic cancer cells in vitro, and decreased tumor growth in vivo. Specifically, Bcl-2, Claspin, METTL3 and YTHDF3 were identified as the potential targets of celastrol treatment in pancreatic cancer cells. Moreover, our results indicated that celastrol treatment downregulated METTL3 and decreased m^6^A levels of Claspin and Bcl-2 mRNA, leading to the degradation of Claspin and Bcl-2 mRNA in pancreatic cancer cells. Furthermore, we revealed that celastrol treatment downregulated Claspin and Bcl-2, at least in part, in an m^6^A-YTHDF3-mediated manner in pancreatic cancer cells.

**Conclusion:**

Our study highlighted a novel mechanism underlying celastrol-induced cellular proliferation inhibition and apoptosis in pancreatic cancer cells via m^6^A-YTHDF3-mediated downregulation of Claspin and Bcl-2.

**Supplementary Information:**

The online version contains supplementary material available at 10.1007/s12672-023-00838-5.

## Introduction

Pancreatic cancer is one of the deadliest cancers worldwide and its mortality has increased considerably, especially in developed countries [1]. Radical surgical resection, chemotherapy, and radiation are frequently employed strategies for the early stage treatment of f patients with pancreatic cancer [2]. Unfortunately, vast majority of patients with pancreatic cancer are frequently identified at advanced stages, and the typical patient lifetime was less than a year [3, 4]. Despite many advances in the combination of gemcitabine with other chemotherapy drugs that may provide a limited increase in survival but with severe side effects, the development of more efficient and less harmful treatments for pancreatic cancer patients is of significant therapeutic importance.

Celastrol has been demonstrated to exhibit potential anticancer activity in a broad range of cancers via various mechanisms, such as inducing cellular apoptosis, inhibiting cell cycle progression and cell proliferation, targeting tumor inflammatory environment, and suppressing cell invasion and metastasis [5–8]. It has also been shown the anti-pancreatic cancer activity of celastrol by up-regulating DDIT3 and ATF3 and down-regulating RRM2 and MCM4 [8], however, the role of celastrol in anti-pancreatic cancer remains largely elusive. Accumulating evidence indicates that *N*^6^-methyladenosine (m^6^A) modification in RNAs is a key epigenetic regulation for gene expression and is connected to the progression of numerous diseases, especially in human cancers [9, 10]. Dysregulation of RNA m^6^A was linked to the occurrence and development of pancreatic cancer [11], however, the underlying regulatory mechanisms involved in pancreatic cancer remain poorly understood. Although the pharmacological anticancer activity of celastrol against pancreatic cancer was well-documented [12, 13], it remains to be evaluated whether celastrol plays an anti-pancreatic cancer role through modulating RNA m^6^A modification.

In the present study, we demonstrated that celastrol treatment induced cellular proliferation inhibition, cell cycle arrest, and apoptosis of the pancreatic cancer cells in vitro and suppressed tumor growth in vivo. Notably, we found that celastrol treatment mediated the reduction of m^6^A levels of Claspin and Bcl-2 mRNA by downregulating METTL3, leading to the degradation of Claspin and Bcl-2 mRNA in pancreatic cancer cells. Further study revealed that celastrol treatment downregulated Claspin and Bcl-2 in an m^6^A-YTHDF3-mediated manner, which providing a novel mechanism for the therapeutic application of celastrol in pancreatic cancer.

## Materials and methods

### Cell culture, transfection and reagents

Human pancreatic cancer cell lines PANC-1, SW1990, AsPC-1 and BxPC-3, and normal pancreatic cell line (HPDE6C) were obtained from Cell Bank of the Chinese Academy of Sciences (Shanghai, China), and cultured in RPMI 1640 supplemented with 10% fetal bovine serum (FBS, Gibco, Carlsbad, CA, USA) and 1% penicillin–streptomycin. Transfection of siRNA targeting human Claspin (si-Claspin sense 5ʹ-UUGGCCACUGAUUUCAAUUdTdT-3ʹ, and antisense 5ʹ-AAUUGAAAUCAGUGGCCAA dTdT-3ʹ) and a control (si-NC) obtained from RiboBio Co., Ltd. (Guangzhou, China) was performed by using the riboFect^™^ CP Transfection Kit (RiboBio Co., Ltd.) according to the manufacturer’s instructions. For Claspin, METTL3 or YTHDF3 overexpression, pcDNA3.1-FLAG-Claspin (Addgen, USA), pEnter-METTL3, pEnter-YTHDF3 (ViGene Biosciences Inc.) or vector controls were used to transfected into the indicated cells by using jetPRIME transfection reagent kit (Polyplus, France) following the manufacturer’s protocols. Celastrol (HPLC = 99.65%) was purchased from MedChemExpress (MCE, Shanghai, China), and dissolved in dimethyl sulfoxide. Cell Counting Kit-8 (CCK-8) was purchased from Vazyme Biotech Co., Ltd (Nanjing, China). Cell-Light^™^ EdU Apollo in Vitro Kit was purchased from RiboBio Co., Ltd. (Guangzhou, China). DNA Content Quantitation kit were purchased from Solarbio Science & Technology (Beijing, China). Annexin V-FITC/PI kit were purchased from Servicebio (Wuhan, China). The antibodies against METTL3 (#86132), Claspin (#2800), YTHDF3 (#24206), GAPDH (#51332), m^6^A (#56593), and HRP-coupled anti-rabbit IgG (#7074) were purchased from Cell Signaling Technology (Danvers, MA, USA). The polyclonal antibody against Bcl-2 (GB113375) was purchased from Servicebio (Wuhan, China).

### Cell viability, cell proliferation and colony formation assays

For CCK-8 assay, 100 µl of the suspension of AsPC-1 or BxPC-3 cells was inoculated into a 96-well plate at a density of 1 × 10^4^ cells/well with six duplications. After 48 h incubation in the complete medium with different concentrations of celastrol, 10 µl of CCK-8 reagent was added into each well followed by incubation for 45 min at 37 ℃ in the incubator. The optical density at 450 nm was captured by a microplate reader. Cell proliferation assay was performed using Cell-Light^™^ EdU Apollo in Vitro Kit according to the manufacturer’s instructions. Briefly, AsPC-1 and BxPC-3 cells were seeded into a 96-well plate at a density of 1 × 10^4^ cells/well with 4 duplications, followed by incubation for 48 h with the indicated concentrations of celastrol. After the EdU labeling, apollo staining, and DNA staining, images were captured under an inverted fluorescence microscope, and the ratio of EdU positive cells was analyzed from five random fields at 100 × magnification. For colony formation assay, AsPC-1 and BxPC-3 cells (100 cells/well) were plated on a 12-well plate with 4 duplications and treated with the indicated concentrations of celastrol for 14 day, the visible colonies were fixed with 4% paraformaldehyde, staining with 1% crystal violet, and analyzed.

### Cell cycle and apoptosis analysis

AsPC-1 and BxPC-3 cells were inoculated in the 6-well plates, followed by incubation with the indicated concentrations of celastrol at 37 ℃ for 48 h. Cell cycle progression and cellular apoptosis were detected by using DNA Content Quantitation kit and Annexin V-FITC/PI kit following the manufacturer’s instructions, respectively, and analyzed by flow cytometry.

### Subcutaneous xenograft assay

Athymic nude mice (BALB/C-nu/nu, 4–6 weeks old, male) were purchased from Changzhou Cavens Laboratory Animal Co., Ltd. (production license No.: SCXK (Su) 2021–0013). All animal procedures were conducted following the institutional ethical requirements and were approved by the Yangzhou University School of Medicine Committee for the Use and Care of Animals. AsPC-1 cells suspended in 100 μl of PBS (2 × 10^6^ cells/100 μl) were injected subcutaneously into the lateral flank of the mice, which were randomly divided into solvent group (n = 6), 1.0 mg/kg of celastrol group (n = 6) and 3.0 mg/kg of celastrol group (n = 6). The administration of celastrol was performed by intraperitoneal injection into tumor-bearing mice every 2 day after 10 day inoculation. The tumor sizes were monitored with calipers every 5 days, and the tumor volume was calculated with the formula: Volume (mm^3^) = 1/2 × length × width^2^. At the end of the experiments, all mice were sacrificed under anesthesia, and the tumors were removed. The tumor weight was noted, and some of the tumor tissues were embedded in paraffin after fixing in formalin. Consecutive sections at 5 µm intervals were used for hematoxylin and eosin (H&E) staining, immunohistochemical staining, or TUNEL apoptosis assay.

### Immunohistochemistry and TUNEL apoptosis assay

After dewaxing, hydration, inactivation of endogenous peroxidases and antigen retrieval, tissue sections were incubated with the primary antibody overnight at 4 ℃, followed by incubation with the secondary antibody at room temperature for 1 h, and then counterstained with H&E. The positive immunohistochemically staining rate score was defined as 0, 1, 2, 3, and 4 represented the percentage of positive stained areas of 0%, 1 – 10%, 11 – 50%, 51 – 80%, and 81 – 100%, respectively. The staining intensity was scored as 0 (negative), 1 (weak), 2 (moderate), or 3 (strong). The final protein expression score was the result of these two indicators. TUNEL apoptosis assay was performed to analyze the apoptosis in the tumor tissue sections using the One Step TUNEL Apoptosis Assay Kit (Servicebio, China), following the manufacturer’s guideline.

### RNA sequencing (RNA-seq) and reverse transcription and quantitative real-time polymerase chain reaction (RT-qPCR)

RNAiso Plus (Takara Biotechnology, Co., Ltd., Dalian, China) was used to extract total RNA from cell samples after incubation with or without celastrol, and RNA-seq analysis was conducted by Wuhan igenebook Co., Ltd. (Wuhan, China). For RT-qPCR, cDNA was reverse transcribed by using HiScript II 1st Strand cDNA Synthesis Kit (+gDNA wiper) (Vazyme, Nanjing, China) and qPCR was performed by using the AceQ Universal SYBR qPCR Master Mix (Vazyme, Nanjing, China) on a LightCycler 96 System (Roche, Switzerland). The primers synthesized by Tsingke Biological Technology (Nanjing, China) were as follows: Claspin forward, 5ʹ-ATGCTTCCCAGATGGACTTG-3ʹ and reverse, 5ʹ-AGCCACTGCTCTCG TTCAAT-3ʹ; Bcl-2 forward, 5ʹ-GGTGGGGTCATGTGTGTGG-3ʹ and reverse, 5ʹ-CGGT TCAGGTACTCAGTCATCC-3ʹ; METTL3 forward, 5ʹ-AAGCTGCACTTCAGACGA AT-3ʹ and reverse, 5ʹ-GGAATCACCTCCGACACTC-3ʹ; YTHDF3 forward, 5ʹ-TGACAA CAAACCGGTTACCA-3ʹ and reverse, 5ʹ-TGTTTCTATTTCTCTCCCTACGC-3ʹ; GAPDH forward, 5ʹ-GCACCGTCAAGGCTGAGAAC-3ʹ and reverse, 5ʹ-TGGTGAAGAC GCCAGTGGA-3ʹ. Relative target gene expression was quantitated by the 2^−ΔΔCT^ method with the normalization to GAPDH.

### Western blotting

Total protein was extracted from the samples using RIPA buffer containing protease and phosphatase inhibitors (New Cell & Molecular Biotech Co. Ltd, Suzhou, China). After quantification of protein with the BCA method, an equal amount of protein samples was subjected to 10% SDS-PAGE, followed by transferred onto the PVDF membrane (Millipore, USA). The blots were probed with the indicated primary antibodies at 4 ℃ overnight, and then incubated with HRP-coupled secondary antibodies at room temperature for 1 h. The immunoblots were developed in a Tanon 5200 Imaging System by using NcmECL Ultra (New Cell & Molecular Biotech Co. Ltd, Suzhou, China) according to the manufacturer's instructions.

### ***RNA dot blot assay and quantification of RNA total m***^***6***^***A modification assay***

Total RNA extracted as described above was placed at 70 ℃ for 5 min, and then chilled on ice immediately. The samples were double diluted and spotted in duplicate onto the Amersham Hybond-N + membranes, followed by ultraviolet crosslinked to the membranes. One of the membranes was stained with 0.02% methylene blue in 0.3 M sodium acetate (pH 5.2) as the loading control. After being blocked in 5% BSA in TBST for 1 h, the other membrane was incubated with anti-m^6^A antibody at 4 ℃ overnight, followed by incubation with the HRP-coupled goat anti-rabbit IgG for 1 h at room temperature. After extensive washing, dot blots were visualized by NcmECL Ultra. For quantification of the total levels of RNA m^6^A in the indicated cells, EpiQuik^™^ m^6^A RNA Methylation Quantification Kit (Epigentek, Farmingdale, NY, USA) was performed according to the manufacturer’s protocol. The m^6^A levels were recorded colorimetrically at a wavelength of 450 nm and then analyzed based on the standard curve.

### Methylated RNA immunoprecipitation (MeRIP) assay

RNAiso Plus was used to extract total RNA from pancreatic cancer cells after 48 h incubation with or without celastrol. MeRIP was conducted using a Magna MeRIP^™^ m^6^A Kit (Millipore, MA, USA) in accordance with the manufacturer’s instructions. The m^6^A-precipitated RNA was harvested and purified, and m^6^A modification in Claspin and Bcl-2 mRNA was evaluated by RT-qPCR (MeRIP-qPCR).

### RNA immunoprecipitation (RIP) assay

The RIP assay was performed using a Magna RIP RNA-Binding Protein Immunoprecipitation Kit (Millipore, MA, USA) according to the manufacturer’s instructions. The RNAs in the immunoprecipitates and input were purified with phenol: chloroform: isoamyl alcohol and then subjected to analyze by RT-qPCR (RIP-qPCR).

### RNA stability and protein stability assays

After incubation with or without celastrol for 48 h, AsPC-1 and BxPC-3 cells inoculated in 6-well plates were treated with a final concentration of 2 μg/ml of actinomycin D (MCE, HY-17559, Shanghai, China) for the indicated times, and total RNA was extracted for RT-qPCR to evaluate the mRNA stability of Claspin and Bcl-2. To evaluate protein stability, AsPC-1 and BxPC-3 cells inoculated in 6-well plates were incubated with or without celastrol for 48 h, and then a final concentration of 10 μM of cycloheximide (MCE, HY-12320, Shanghai, China) was added into each well. After incubation at the indicated times, treated cells were harvested, and total protein was extracted for Western blotting analysis.

### Statistical Analysis

All data were analyzed with SPSS20.0 software and presented as the means ± standard deviation (SD) from at least three independent experiments. Data were analyzed by Student’s *t*-test between two group comparisons and by one-way ANOVA for multiple group comparisons. *P*-values less than 0.05 was considered statistically significant.

## Results

### Effect of celastrol on cell viability, cell proliferation, cell cycle progression and apoptosis in AsPC-1 and BxPC-3 cells

**Fig. 1:**
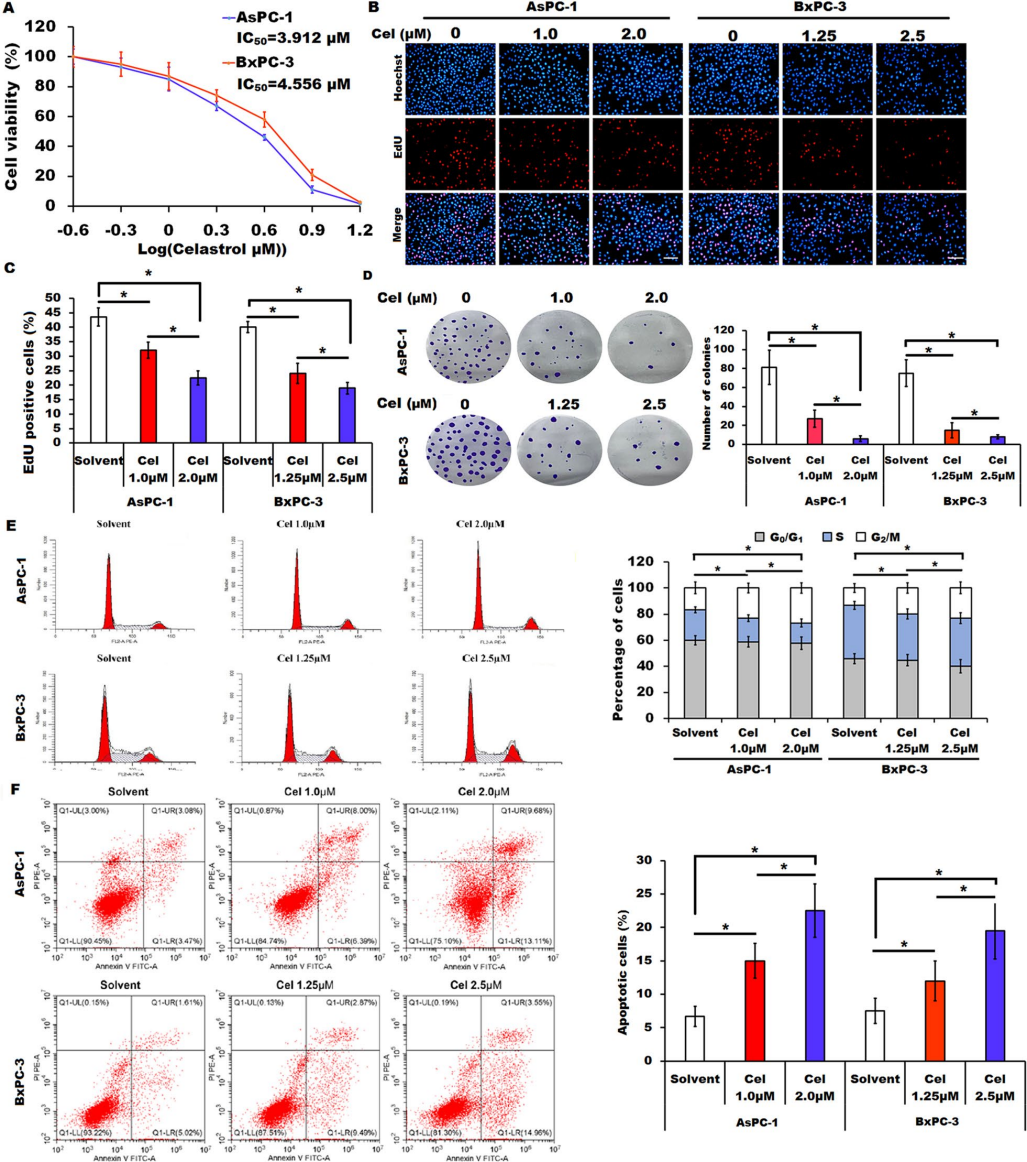
Effect of celastrol treatment on the proliferation and apoptosis of the pancreatic cancer cells. **A** AsPC-1 and BxPC-3 cells were incubated with indicated doses of celastrol for 48 h, cell viability was assessed by CCK-8 assay. **B**, **C** Cell proliferation of AsPC-1 and BxPC-3 cells in the presence or absence of the indicated doses of celastrol (Cel) was detected by EdU assay. Scale bars = 100 μm. **D** Colony formation assay for AsPC-1 and BxPC-3 cells in the presence or absence of the indicated doses of celastrol (Cel) was shown. **E** Cell cycle progression was analyzed by flow cytometry in AsPC-1 and BxPC-3 cells in the presence or absence of the indicated doses of celastrol (Cel). **F** Cellular apoptosis of AsPC-1 and BxPC-3 cells in the presence or absence of the indicated doses of celastrol (Cel) was analyzed by flow cytometry. All data were presented as the means ± SD from three independent experiments. *P < 0.05

Celastrol has been shown the potential anticancer activity in various cancers, but its underlying mechanisms involved in anti-pancreatic cancer remain largely unknown. To evaluate the IC_50_ values of celastrol to the pancreatic cancer cell lines AsPC-1 and BxPC-3, we first applied CCK-8 assay to assess the effect of celastrol on cell viability. After 48 h incubation with the indicated concentrations of celastrol (0, 0.25, 0.5, 1.0, 2.0, 4.0, 8.0, or 16.0 µM), both AsPC-1 and BxPC-3 cells showed the reduction of cell viability in a dose-dependent manner. The IC_50_ values for celastrol against AsPC-1 and BxPC-3 cells were 3.912 µM and 4.556 µM, respectively (Fig. 1A). Based on the results, we selected 1.0 µM and 2.0 µM of celastrol for AsPC-1 cells and 1.25 µM and 2.5 µM of celastrol for BxPC-3 cells to further elicit underlying mechanisms in pancreatic cancer cells. Results from the EdU assay showed that celastrol treatment decreased the proliferation of both AsPC-1 and BxPC-3 cells when compared to the indicated control cells (Fig. 1B and C). Meanwhile, an obvious inhibition in the colony formation of both AsPC-1 and BxPC-3 cells induced by celastrol was observed by the colony formation assay (Fig. 1D). Furthermore, data from flow cytometry analysis showed that celastrol treatment induced G_2_/M phase arrest and enhanced cellular apoptosis in both AsPC-1 and BxPC-3 cells (Fig. 1E and F). Collectively, these data suggested that celastrol treatment decreased cell proliferation and induced cell cycle arrest and apoptosis of the pancreatic cancer cells, which was consistent with other studies [14, 15].

### *Celastrol inhibited tumor growth *in vivo

**Fig. 2:**
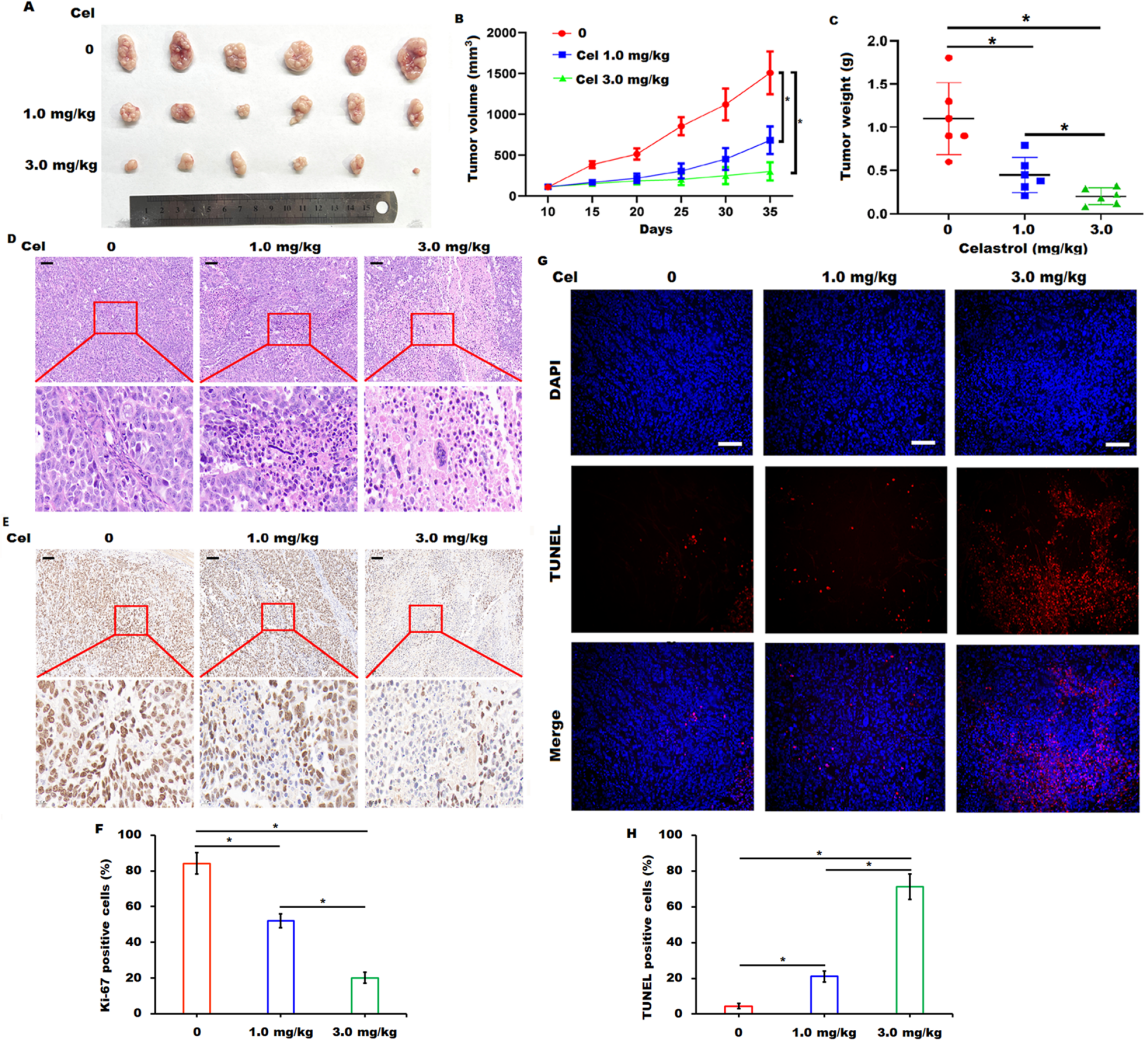
Celastrol administration impaired the xenograft tumor growth in vivo. **A-C** Subcutaneous xenograft tumors were collected (**A**), and tumor volume (**B**) and tumor weight (**C**) were recorded in indicated celastrol (Cel) administration groups. n = 6. **D** Representative H&E staining images in tissue sections of tumors in indicated celastrol (Cel) administration groups were shown. Scale bars = 100 μm. **E**, **F** Representative immunohistochemical staining for ki-67 in tissue sections of tumors in indicated celastrol (Cel) administration groups were shown. Scale bars = 100 μm. **G**, **H** Cellular apoptosis in tissue sections of tumors in indicated celastrol (Cel) administration groups was analyzed by TUNEL apoptosis assay. Scale bars = 100 μm. Data were presented as the means ± SD, *P < 0.05

To further validate whether celastrol administration inhibited tumor growth in vivo*,* the tumor xenograft models were established by subcutaneously injecting AsPC-1 cells into nude mice. As shown in Fig. 2A–C, celastrol administration either in the 1.0 mg/kg of celastrol group or the 3.0 mg/kg of celastrol group led to a significant reduction in tumor volume and tumor weight. Histological analysis using H&E staining demonstrated that the tumor cells showed brisk mitotic activity and grew with little foci of necrosis in control group, whereas the tumor tissues displayed many wide areas of necrosis in the administration of celastrol groups, especially in the 3.0 mg/kg of celastrol group (Fig. 2D). Immunohistochemical staining and TUNEL assay further confirmed that celastrol administration obviously decreased the number of Ki-67 positive cells and increased the apoptotic cells in xenograft tumor tissues, especially in the 3.0 mg/kg of celastrol group (Fig. 2E and F). The above data suggested that the reduction in tumor growth induced by celastrol administration probably accounted for the celastrol-induced cell proliferation inhibition and apoptosis in pancreatic cancer.

### Claspin, Bcl-2, METTL3 and YTHDF3 were identified as the potential targets of celastrol

**Fig. 3:**
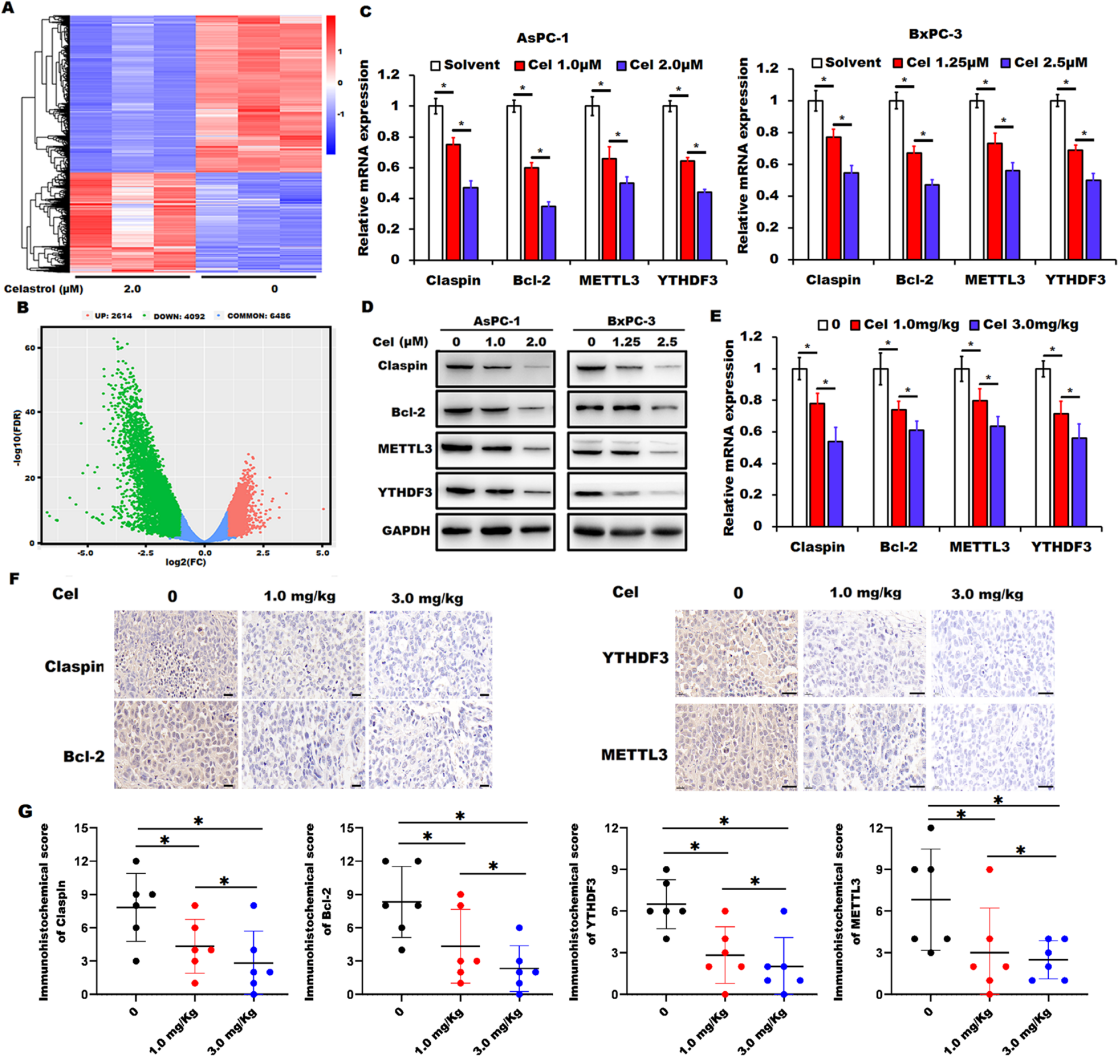
Celastrol treatment regulated the expression of Claspin, Bcl-2, METTL3, and YTHDF3 in pancreatic cancer cells. **A**, **B** Clustered heatmap of the differentially expressed genes in AsPC-1 cells in the presence or absence of celastrol (both in triplicate) by RNA-seq analysis (**A**), and the volcano plots of the differentially expressed genes were shown (**B**). **C**, **D** RT-qPCR (**C**) and Western blotting (**D**) were applied to validate the expression of Claspin, Bcl-2, METTL3, and YTHDF3 in AsPC-1 and BxPC-3 cells in the presence or absence of celastrol (Cel). GAPDH was used as a loading control. **E** RT-qPCR was performed to validate the expression of Claspin, Bcl-2, METTL3, and YTHDF3 in xenograft tumor tissues in indicated celastrol (Cel) administration groups. **F**, **G** Representative immunohistochemical staining and immunohistochemical score for Claspin, Bcl-2, METTL3, and YTHDF3 in tissue sections of tumors from indicated celastrol (Cel) administration groups was shown. Data were shown as the means ± SD from three independent experiments, *P < 0.05. Scale bars = 20 µm

To identify the potential targets involved in celastrol-induced cell proliferation inhibition and apoptosis in pancreatic cancer cells, we employed RNA-seq to illustrate the transcriptional alterations in celastrol-treated AsPC-1 cells. We found that 2614 up-regulated genes and 4092 down-regulated genes were differentially expressed in celastrol-treated AsPC-1 cells (Fig. 3A and B). Among the differentially expressed genes, *CLSPN* (encoding Claspin) and *BCL2* (encoding Bcl-2), two important regulators of cell proliferation and apoptosis [16, 17], and *METTL3* and *YTHDF3*, two well-characterized regulators related to RNA m^6^A modification [18, 19], showed a substantial alteration in celastrol-treated AsPC-1 cells. Consistent with the data from RNA-seq, the results from RT-qPCR and Western blotting analysis confirmed that celastrol treatment significantly downregulated the expression of Claspin, Bcl-2, METTL3, and YTHDF3 at the mRNA and protein levels in both AsPC-1 and BxPC-3 cells when compared to those in the indicated control cells (Fig. 3C and D). In addition, RT-qPCR and immunohistochemical staining also showed that celastrol administration downregulated Claspin, Bcl-2, METTL3, and YTHDF3 in the xenograft tumor tissues (Fig. 3E–G). Collectively, our findings indicated that Claspin, Bcl-2, METTL3, and YTHDF3 might be the potential targets of celastrol treatment in pancreatic cancer cells.

### Celastrol treatment induced cell proliferation inhibition and apoptosis probably by downregulating Claspin

**Fig. 4:**
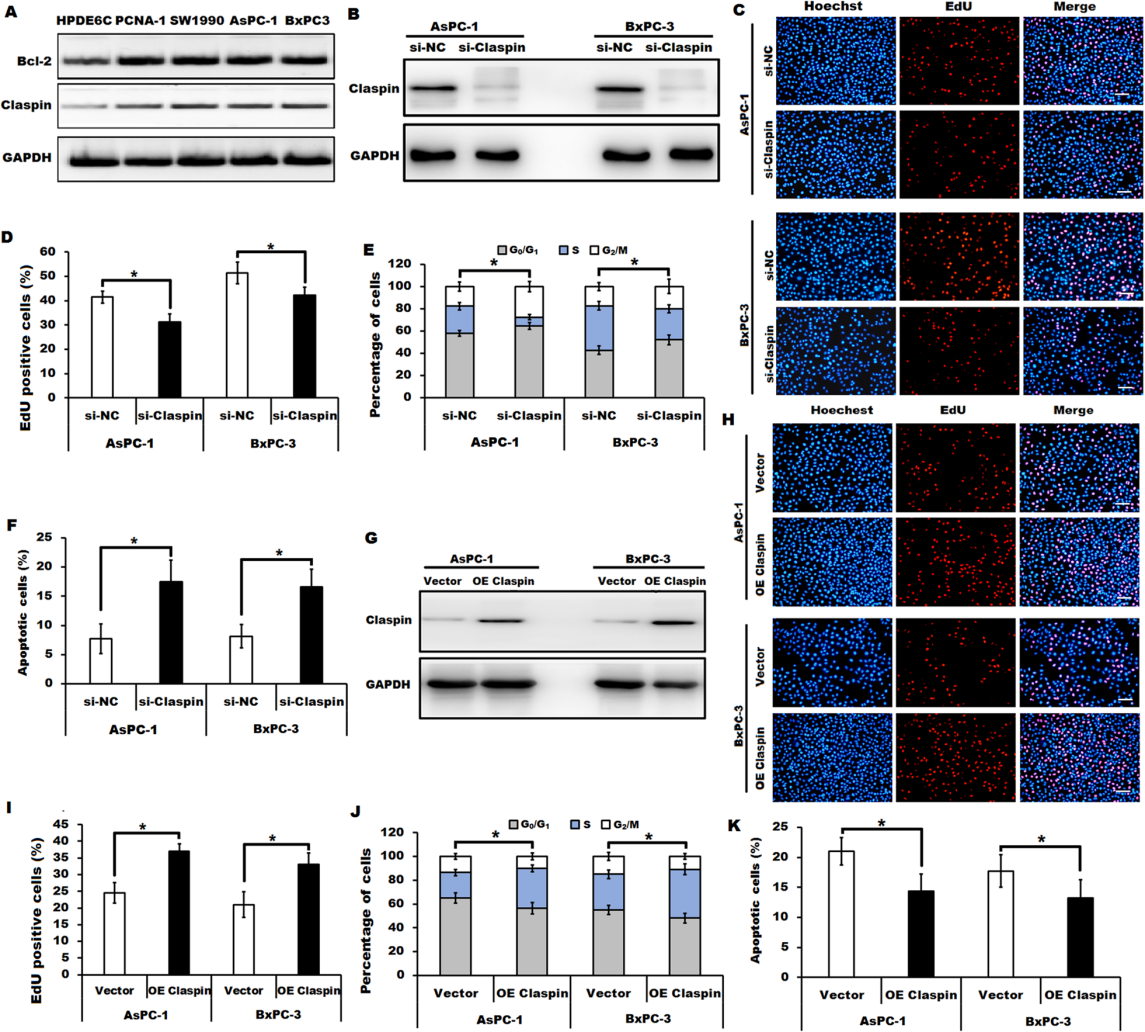
Celastrol induced cell proliferation inhibition and apoptosis by downregulating Claspin in pancreatic cancer cells. **A** Western blotting was performed to evaluate the expression of Bcl-2 and Claspin in pancreatic cancer cell lines (PANC-1, SW1990, AsPC-1 and BxPC-3) and the normal pancreatic cells (HPDE6C7). GAPDH served as loading control. n = 3. **B** The knockdown efficiency of Claspin in AsPC-1 and BxPC-3 cells transfected with si-Claspin or the control (si-NC) was determined by Western blotting. GAPDH served as loading control. n = 3. **C**, **D** Cell proliferation of AsPC-1 and BxPC-3 cells transfected with si-Claspin or si-NC was detected by EdU assay. Scale bars = 100 μm. **E**, **F** Representative cell cycle analysis (**E**) and apoptotic analysis (**F**) in Claspin knocking down cells (si-Claspin) and the indicated control cells (si-NC) were shown. **G** Overexpression of Claspin in celastrol (2.0 µM)-treated AsPC-1 and celastrol (2.5 µM)-treated BxPC-3 cells after transfection with the vector encoding Claspin (OE Claspin) or control (vector) was confirmed by Western blotting. GAPDH served as loading control. n = 3. **H**, **I** Representative EdU assay in Claspin overexpressing cells (OE Claspin) and the indicated control cells (vector) after incubation with celastrol (2.0 µM for AsPC-1 and 2.5 µM for BxPC-3 cells). Scale bars = 100 μm. **J and K** Representative cell cycle analysis (**J**) and apoptotic analysis (**K**) in Claspin overexpressing cells (OE Claspin) and the indicated control cells (vector) after incubation with celastrol (2.0 µM for AsPC-1 and 2.5 µM for BxPC-3 cells) were shown. All data were shown as the means ± SD from three independent experiments, *P < 0.05

It is notable that the elevated abundance of Claspin and Bcl-2 was shown in human pancreatic cancer cell lines (PANC-1, SW1990, AsPC-1, and BxPC-3) when compared to those in normal pancreatic cells (HPDE6C) as measured by Western blotting (Fig. 4A). Based on the well illustration of the role of Bcl-2 in cancer progression [20, 21], we focused on the role of Claspin in celastrol-treated pancreatic cancer cells. We confirmed that silencing of Claspin by transfection with specific siRNA against Claspin (si-Claspin) resulted in the inhibition of cell proliferation and cell cycle progression, and the promotion of apoptosis in both AsPC-1 and BxPC-3 cells (Fig. 4B-F). Furthermore, we revealed that overexpression of Claspin by transfection with pcDNA3.1-FLAG-Claspin attenuated the suppression of cell proliferation, cell cycle progression and the induction of apoptosis in celastrol-treated AsPC-1, and BxPC-3 cells when compared to those in the indicated vector controls as determined by EdU incorporation assay and flow cytometry (Fig. 4G-K). Therefore, we supposed that celastrol treatment induced cell proliferation inhibition and apoptosis probably by downregulating Claspin in pancreatic cancer cells.

### ***Celastrol treatment down-regulated m***^***6***^***A modification of Claspin and Bcl-2 mRNA***

**Fig. 5:**
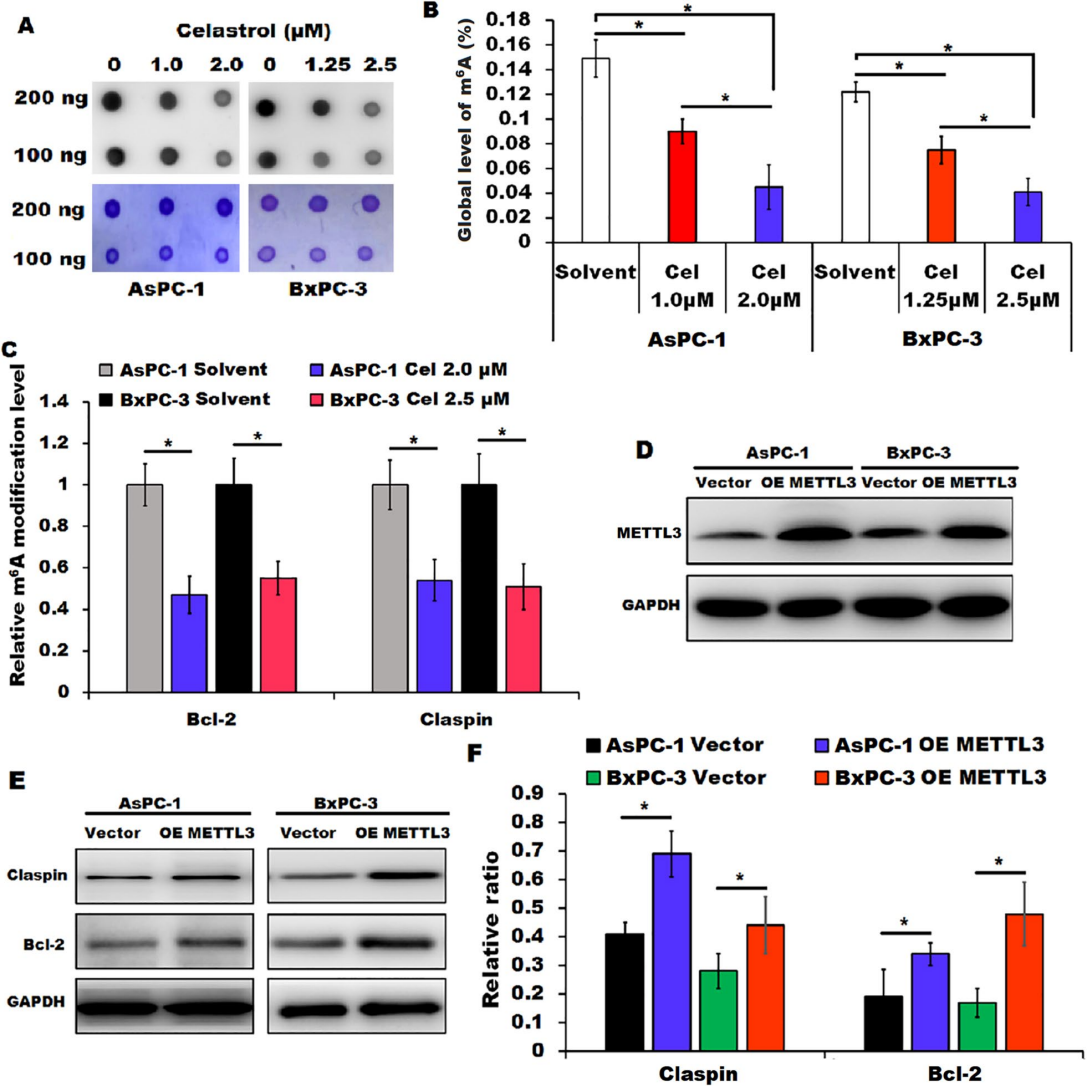
Celastrol down-regulated m^6^A modification of Claspin and Bcl-2 mRNA in pancreatic cancer cells.** A** RNA dot blot assay was performed to detect the global RNA m^6^A levels in AsPC-1 and BxPC-3 cells in the presence or absence of celastrol (upper), and methylene blue staining was shown as loading control (down). n = 3. **B** RNA m^6^A quantitative detection assay was performed to detect the overall RNA m^6^A levels in AsPC-1 and BxPC-3 cells in the presence or absence of celastrol. **C** MeRIP-qPCR was performed to evaluate the m^6^A modification in Claspin and Bcl-2 mRNA in AsPC-1 and BxPC-3 cells in the presence or absence of celastrol (Cel). **D** Overexpression of METTL3 in celastrol (2.0 µM)-treated AsPC-1 and celastrol (2.5 µM)-treated BxPC-3 cells after transfection with the vector encoding METTL3 (OE METTL3) or vector control (vector) was confirmed by Western blotting. GAPDH served as loading control. n = 3. **E**, **F** Expression of Claspin and Bcl-2 in METTL3 overexpressing and control cells after incubation with celastrol (2.0 µM for AsPC-1 and 2.5 µM for BxPC-3 cells) was detected by Western blotting. GAPDH served as loading control. All data were shown as the means ± SD from three independent experiments, *P < 0.05

Considering that METTL3, a core RNA methyltransferase, can catalyze the formation of RNA m^6^A, we next evaluated whether celastrol treatment regulated the global levels of RNA m^6^A in pancreatic cancer cells by RNA m^6^A dot blot assay and quantification of RNA total m^6^A modification assay, respectively. We found that the overall levels of RNA m^6^A were decreased in celastrol-treated both AsPC-1 and BxPC-3 cells in comparison with those in the indicated control cells (Fig. 5A and B). We then performed MeRIP-qPCR to evaluate whether celastrol treatment regulated the levels of m^6^A modification in Claspin and Bcl-2 mRNA. As expected, celastrol treatment resulted in a significant decrease of the level of the m^6^A modification within Claspin and Bcl-2 mRNA (Fig. 5C). In addition, we overexpressed the METTL3 in AsPC-1 and BxPC-3 cells by transfection with pEnter-METTL3 and demonstrated that celastrol-induced downregulation of Claspin and Bcl-2 was partially rescued by METTL3 overexpression (Fig. 5D-F), suggesting that METTL3 participated in celastrol-mediated regulation of Claspin and Bcl-2 in pancreatic cancer cells. Based on these data, it can be concluded that celastrol treatment decreased the global RNA m^6^A modification, especially reduced the mRNA m^6^A modification of Claspin and Bcl-2 probably by downregulating METTL3, leading to the downregulation of Claspin and Bcl-2 in pancreatic cancer cells.

### ***Celastrol treatment reduced the expression of Claspin and Bcl-2 in an m***^***6***^***A-YTHDF3-mediated manner***

**Fig. 6:**
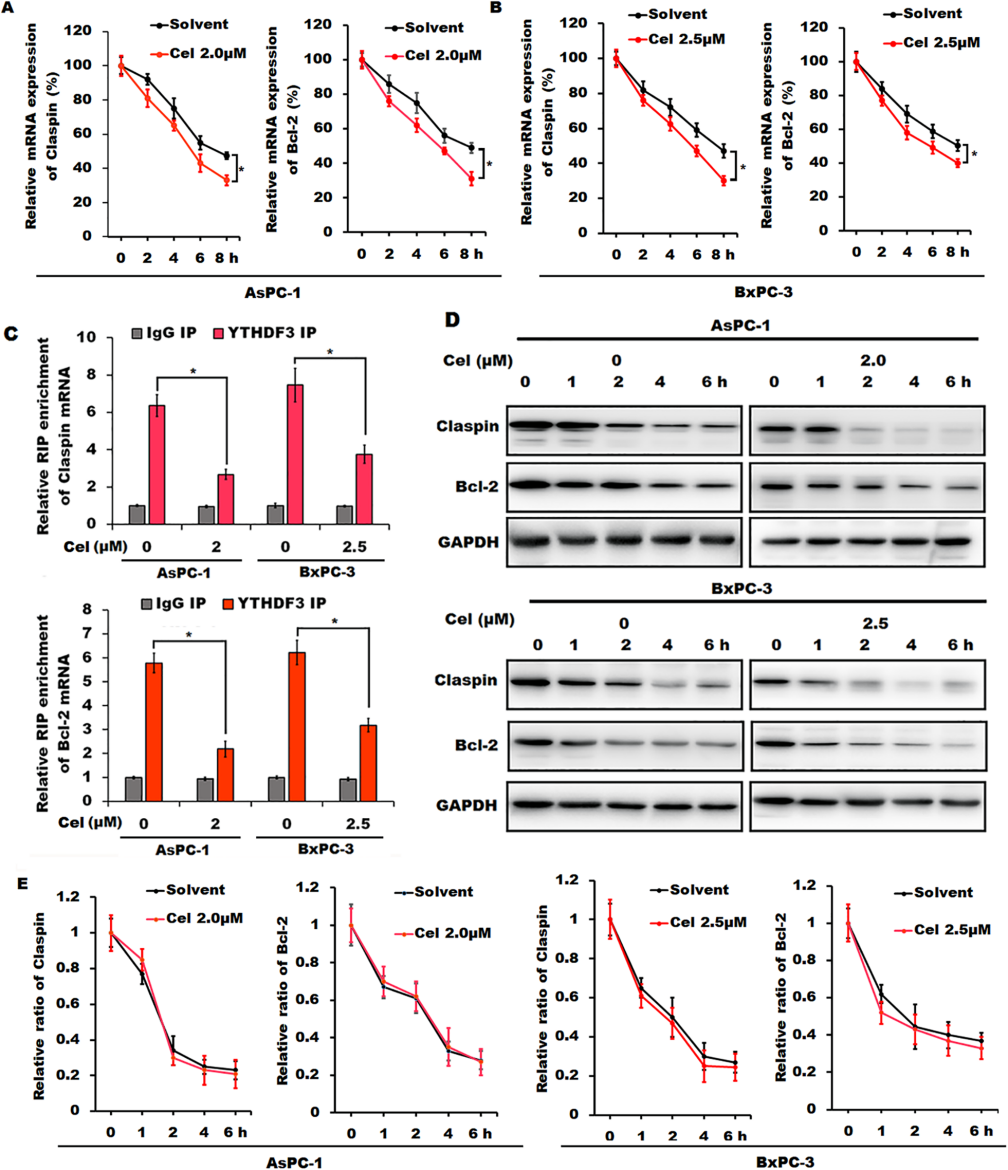
Celastrol down-regulated Claspin and Bcl-2 expression in an m^6^A-YTHDF3 mediated pattern in pancreatic cancer cells. **A**, **B** The relative levels of Bcl-2 or Claspin mRNA in celastrol-treated both AsPC-1 (**A**) and BxPC-3 cells (**B**) after incubation with actinomycin D (2 μg/ml) for the indicated times (normalized to 0 h) were determined by RT-qPCR. **C** RIP-qPCR assay was performed to evaluate the interaction between YTHDF3 and the mRNA of Claspin or Bcl-2 in AsPC-1 and BxPC-3 cells in the presence or absence of celastrol. **D**, **E** The relative levels of Claspin or Bcl-2 protein in celastrol-treated AsPC-1 and BxPC-3 cells after incubation with cycloheximide for the indicated times were determined by Western blotting (**D**) and quantitatively analyzed (**E**). All data were shown as the means ± SD from three independent experiments, *P < 0.05

**Fig. 7:**
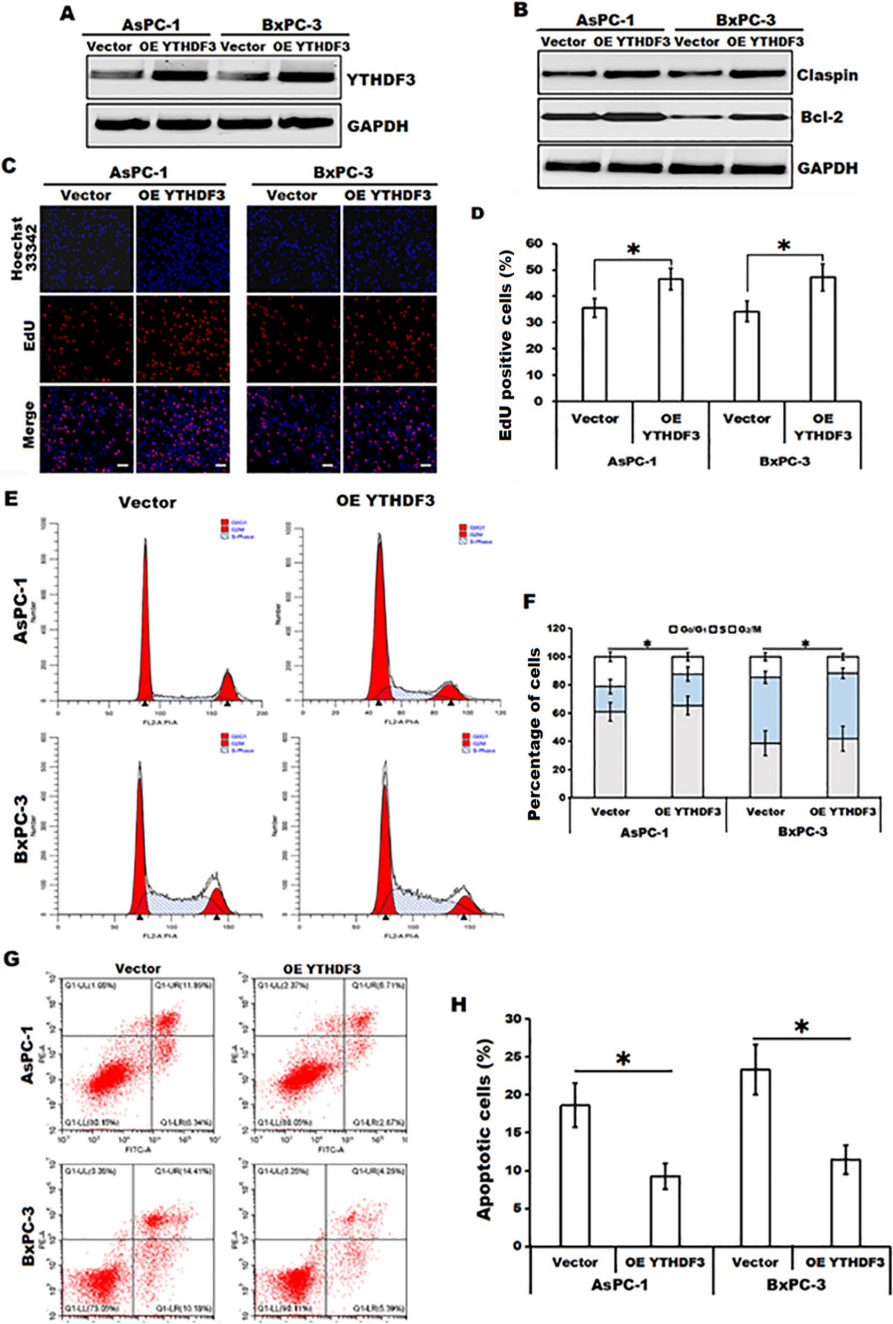
Claspin and Bcl-2 might be the targets of YTHDF3 in celastrol-treated pancreatic cancer cells. **A** Overexpression of YTHDF3 in celastrol (2.0 µM)-treated AsPC-1 and celastrol (2.5 µM)-treated BxPC-3 cells after transfection with the vector encoding YTHDF3 (OE YTHDF3) or vector control (vector) was confirmed by Western blotting. GAPDH served as loading control. n = 3. **B** Expression of Claspin and Bcl-2 in YTHDF3 overexpressing (OE YTHDF3) and control cells (vector) after incubation with celastrol (2.0 µM for AsPC-1 and 2.5 µM for BxPC-3 cells) was evaluated by Western blotting. GAPDH served as loading control. n = 3. **C**, **D** Representative EdU assay in YTHDF3 overexpressing (OE YTHDF3) and control cells (vector) after incubation with celastrol (2.0 µM for AsPC-1 and 2.5 µM for BxPC-3 cells) was shown. Scale bars = 100 μm. **E**, **F** Representative cell cycle analysis in YTHDF3 overexpressing (OE YTHDF3) and control cells (vector) after incubation with celastrol (2.0 µM for AsPC-1 and 2.5 µM for BxPC-3 cells) was shown. **G**, **H** Representative cellular apoptotic analysis in YTHDF3 overexpressing (OE YTHDF3) and control cells (vector) after incubation with celastrol (2.0 µM for AsPC-1 and 2.5 µM for BxPC-3 cells) was shown. All data were shown as the means ± SD from three independent experiments, *P < 0.05

Since RNA m^6^A modification has been shown to play important roles in regulating mRNA splicing, stability, and translation [22], we further investigated whether celastrol treatment affected the mRNA decay of Claspin and Bcl-2 in AsPC-1 and BxPC-3 cells. Data from RNA stability assay showed that the degradation of Claspin and Bcl-2 mRNA was significantly accelerated in celastrol-treated AsPC-1 and BxPC-3 cells after incubation with actinomycin D during the indicated times when compared with those in the indicate control cells (Fig. 6A and B), indicating that celastrol-induced the decrease of the m^6^A modification can trigger the degradation of Claspin and Bcl-2 mRNA. Based on the above analysis of celastrol treatment downregulating YTHDF3 expression, which is one of the m^6^A readers and can bind m^6^A sites to regulate target mRNA stability and protein translation [23], we then performed RIP-qPCR assay to confirm the interaction between YTHDF3 and Claspin or Bcl-2 mRNA. Results from RIP-qPCR analysis showed that both Claspin and Bcl-2 mRNA were enriched by YTHDF3-specific antibody in both AsPC-1 and BxPC-3 cells, whereas these relative enrichments were obviously decreased in celastrol-treated cells (Fig. 6C), indicating that Claspin and Bcl-2 mRNA might be the potential targets of YTHDF3. Moreover, we also evaluated whether celastrol treatment influenced the protein stability or translation efficiency of Claspin and Bcl-2. Data from western blotting analysis demonstrated that there was no significant alteration of the protein stability of Claspin and Bcl-2 between celastrol-treated AsPC-1 and BxPC-3 cells and the indicated control cells (Fig. 6D and E), indicating that celastrol-mediated downregulation of Claspin and Bcl-2 was not due to modulating their protein stability. In addition, we further observed that overexpression of YTHDF3 partially reversed celastrol-induced downregulation of Claspin and Bcl-2, and abrogated the celastrol-mediated suppression of cell proliferation and cell cycle progression, and the induction of apoptosis in celastrol-treated AsPC-1 and BxPC-3 cells when compared to those in the indicated vector control cells (Fig. 7). Collectively, our data clearly indicated that Claspin and Bcl-2 might be the targets of YTHDF3, and celastrol treatment downregulated Claspin and Bcl-2 in an m^6^A-YTHDF3-mediated manner in pancreatic cancer cells.

## Discussion

Celastrol has been revealed to exhibit a wide range of biochemical activities, including anti-inflammatory, anti-diabetes, and anti-autoimmune diseases activities [24]. Increasing evidences have addressed the anticancer activity of celastrol against various types of cancers [3, 4, 25], but its antitumor activity in pancreatic cancer largely remains unknown. In the present study, we demonstrated that celastrol inhibited the malignant proliferation, induced cell cycle arrest and apoptosis of pancreatic cancer cells in vitro, and suppressed tumor growth in vivo, which functionally further confirmed the anti-pancreatic cancer role of celastrol. Mechanistically, we demonstrated that celastrol-induced cellular proliferation inhibition and apoptosis in pancreatic cancer cells by downregulating Claspin and Bcl-2 expression in an m^6^A-YTHDF3-mediated manner. Our research suggested that celastrol might be developed into a promising candidate as a potential therapy option for pancreatic cancern.

To better understand the mechanisms of celastrol-induced cell proliferation inhibition and apoptosis in pancreatic cancer cells, we first performed RNA-seq analysis to identify potential targets in celastrol-treated AsPC-1 cells. There are 6706 differentially expressed genes in celastrol-treated AsPC-1 cells. Consistently, we validated and identified four downregulated genes, *CLSPN*, *BCL2*, *METTL3* and *YTHDF3*, as the potential downstream targets of celastrol in both AsPC-1 and BxPC-3 cells. Claspin and Bcl-2 are important regulators of cell proliferation and apoptosis [16, 17], while METTL3 and YTHDF3 are two well-characterized regulators involved in RNA m^6^A modification [18, 19]. Here, we observed the up-regulation of Claspin and Bcl-2 in pancreatic cancer cell lines when compared with those in normal pancreatic cells. As a member of anti-apoptotic proteins, the role of Bcl-2 in cancer progression has been well illustrated [20, 21], whereas the biological function of Claspin in tumorigenesis remains largely unknown. Claspin has primarily been characterized as an adaptor protein that participated in the modulation of cell cycle progression, DNA damage and repair, and apoptosis [26, 27], however, little is known about the role of Claspin in pancreatic cancer. Herein, we provided evidence that silencing of Claspin in pancreatic cancer cells led to cellular proliferation inhibition and apoptosis. Furthermore, we performed a rescue assay and confirmed that overexpression of Claspin partially rescued celastrol-induced cell proliferation inhibition, cell cycle arrest and apoptosis in celastrol-treated cells. These data indicated that Claspin and Bcl-2 were involved in celastrol-mediated regulation in cell proliferation, cell cycle progression and apoptosis in pancreatic cancer cells.

RNA m^6^A modification has been shown to participate in the progression of pancreatic cancer [11, 28]. As a core RNA m^6^A methyltransferase, METTL3 promotes the formation of RNA m^6^A modification in mammalian cells [29]. In the current study, we demonstrated the downregulation of METTL3 and decrease of the global RNA m^6^A levels in celastrol-treated pancreatic cancer cells. Moreover, we uncovered that overexpression of METTL3 partially reversed celastrol-induced downregulation of Claspin and Bcl-2, which provided the causative link between RNA m^6^A modification and the expression of Claspin and Bcl-2 induced by celastrol treatment in pancreatic cancer cells. We further revealed a significant decrease of m^6^A modification of Claspin and Bcl-2 mRNA in celastrol-treated cells as determined by MeRIP-qPCR. Additionally, we noted that celastrol treatment facilitated the mRNA decay of Claspin and Bcl-2, suggesting that celastrol-mediated decrease of the m^6^A modification might trigger the mRNA degradation of Claspin and Bcl-2 in pancreatic cancer cells.

As one of the m^6^A readers, YTHDF3 is responsible for regulating m^6^A-mediated mRNA stability and translation [30]. In this study, we showed downregulation of YTHDF3 in celastrol-treated pancreatic cancer cells. Then we confirmed the interaction between YTHDF3 and the mRNA of Claspin and Bcl-2 in both AsPC-1 and BxPC-3, while the interaction of YTHDF3 with Claspin mRNA or Bcl-2 mRNA was significantly decreased in celastrol-treated cells. However, our protein stability assay revealed that celastrol treatment had no effect on both Claspin and Bcl-2 protein stability, indicating that downregulation of Claspin and Bcl-2 protein in celastrol-treated cells was not due to the effect of celastrol on their protein stability or translation efficiency. Furthermore, we noted that YTHDF3 overexpression partially rescued celastrol-induced downregulation of Claspin and Bcl-2, and abrogated the celastrol-mediated suppression of cell proliferation and cell cycle progression, and the induction of apoptosis in celastrol-treated in pancreatic cancer cells. Taken together, our data clearly indicated that celastrol treatment downregulated Claspin and Bcl-2, at least in part, in an m^6^A-YTHDF3-mediated manner in pancreatic cancer cells.

In summary, the findings present here provided in vitro and in vivo evidences demonstrating that downregulation of Bcl-2, Claspin, METTL3 and YTHDF3 was involved in celastrol-induced cellular proliferation inhibition, cell cycle arrest and apoptosis in pancreatic cancer cells. We demonstrated that celastrol treatment reduced total RNA m^6^A modification in pancreatic cancer cells by down-regulating METTL3 expression, especially decreased Claspin and Bcl-2 mRNA m^6^A modification, which induced the Claspin and Bcl-2 mRNA decay. We further highlighted a novel mechanism underlying celastrol-induced cellular proliferation inhibition and apoptosis in pancreatic cancer cells by downregulating Claspin and Bcl-2 expression, which was due to the down-regulation of YTHDF3 induced by celastrol treatment and thereby reduced the interaction of YTHDF3 with Claspin and Bcl-2 mRNA.

### Supplementary Information

**Additional file 1. Table S1** Information about the kits.

## Data Availability

The datasets and materials used and/or analyzed during the current study are available from the corresponding author on reasonable request.
